# The Association between Road Traffic Noise Exposure, Annoyance and Health-Related Quality of Life (HRQOL)

**DOI:** 10.3390/ijerph111212652

**Published:** 2014-12-05

**Authors:** Harris Héritier, Danielle Vienneau, Patrizia Frei, Ikenna C. Eze, Mark Brink, Nicole Probst-Hensch, Martin Röösli

**Affiliations:** 1 Swiss Tropical and Public Health Institute, Socinstr. 57, P.O. Box, CH-4002 Basel, Switzerland; E-Mails: harris.heritier@unibas.ch (H.H.); danielle.vienneau@unibas.ch (D.V.); ikenna.eze@unibas.ch (I.C.E.); nicole.probst@unibas.ch (N.P.-H.); 2 University of Basel, Petersplatz 1, CH-4003 Basel, Switzerland; 3 Krebsliga Schweiz, 3001 Bern, Switzerland; E-Mail: patrizia.frei@krebsliga.ch; 4 Federal Office for the Environment, 3003 Bern, Switzerland; E-Mail: mark.brink@bafu.admin.ch

**Keywords:** noise, exposure, annoyance, health indicators, von Zerssen, SF-36, quality of life

## Abstract

The aim of this study is to investigate the relationships between road traffic noise exposure, annoyance caused by different noise sources and validated health indicators in a cohort of 1375 adults from the region of Basel, Switzerland. Road traffic noise exposure for each study participant was determined using modelling, and annoyance from various noise sources was inquired by means of a four-point Likert scale. Regression parameters from multivariable regression models for the von Zerssen score of somatic symptoms (point symptom score increase per annoyance category) showed strongest associations with annoyance from industry noise (2.36, 95% CI: 1.54, 3.17), neighbour noise (1.62, 95% CI: 1.17, 2.06) and road traffic noise (1.53, 95% CI: 1.09, 1.96). Increase in modelled noise exposure by 10 dB(A) resulted in a von Zerssen symptom score increase of 0.47 (95% CI: −0.01, 0.95) units. Subsequent structural equation modelling revealed that the association between physical noise exposure and health-related quality of life (HRQOL) is strongly mediated by annoyance and sleep disturbance. This study elucidates the complex interplay of different factors for the association between physical noise exposure and HRQOL.

## 1. Introduction

Annoyance is one of the numerous health effects related to noise exposure and affects a large share of the population worldwide. Annoyance, often also triggered at low noise levels, has been the focus of previous environmental noise research [1,2]. Numerous studies found a positive exposure-response relationship for annoyance with increasing noise exposure from various sources [3,4,5]. In 2011, the WHO estimated that the share of the European population highly annoyed by road traffic noise at levels >55 dB(A) was 25% [6]. Upon extrapolation, it was estimated that annoyance induces losses in the range of 0.32–3.92 million disability adjusted life years or DALYs/year in the European Union [6].

In recent years, the evidence linking noise exposure and indicators of annoyance-mediated degradation of quality of life has accumulated. Studies have shown marked associations between noise exposure and annoyance with disturbance [2,7,8], reduced wellbeing [2,7] and reduced health-related quality of life (HRQOL) [2,8,9].

According to the Burden of Disease Report of the WHO [6], people annoyed by noise may experience a range of negative responses such as depression, anxiety or exhaustion, thus augmenting stress which is a recognised risk factor for cardiovascular diseases. For this reason, a better understanding of annoyance and its influence on health may help to prevent future health degradation. As stated in the theoretical framework of Stallen [10] and Soames [11], annoyance plays a role in mediating the further development of noise-induced health effects. Indeed, an internal mechanism of appraisal based on a set of non-acoustical factors such as attitude towards the noise source [10] or noise sensitivity [11] modify the annoyance reaction. Thus, subjects lacking the internal resource to overcome noise-induced stress and annoyance are more likely to present signs of health degradation in the long-term, although noise effects on sleep have also been observed in people who are not annoyed by noise [12]. In previous work [13,14,15] structural equation models have been used to disentangle the complex interplay between noise and noise-related variables such as annoyance, sleep disturbance, noise sensitivity and HRQOL.

Further, the association between annoyance and any health outcome may be modified by factors such as sleep deprivation or body mass index (BMI). Indeed, the recent study of Sørensen *et al.* [16] indicate that BMI may play a role in noise induced health effects. A recent analysis using the same data as the present paper found that the association between road traffic noise and sleep was modified by gender [17].

The present study investigated the association between road traffic noise exposure and annoyance, and health indicators. It is based on a cohort study on HRQOL in relation to environmental factors conducted in the Basel area in Switzerland [18]. Whereas a previous analysis focussed on noise induced sleep effects [12], the present paper addresses the interplay between noise, annoyance to noise, sleep disturbance and HRQOL, and explores potential modifying factors such as socio-demographic factors, BMI, comorbidity and noise exposure level. We further investigate the importance of annoyance and sleep disturbance as mediators of the association between physical noise and HRQOL indicators by structural equation modelling (SEM).

## 2. Methods

We used data from the QUALIFEX study (HRQOL and radio frequency electromagnetic field (RF-EMF) exposure: prospective cohort study), which focussed on health effects of RF-EMF and various other environmental exposures [18,19]. In May 2008, questionnaires entitled “environment and health” were sent to 4000 randomly selected residents from the region of Basel (2000 each from the cantons of Basel-City and Basel-Country), Switzerland, aged between 30 and 60 years. Reasons of non-eligibility in the cohort were severe disabilities, death, incorrect addresses (no possible matching with modelled noise exposure), absence during the time of the survey, and problems understanding the questionnaire due to language. After one year, a follow-up was conducted by sending the same questionnaire to the respondents of the baseline survey. Ethical approval for the conduct of the study was received from the ethics committee of Basel on 19 March 2007 (EK: 38/07).

### 2.1. Outcome Variables

The questionnaire consisted of a battery of validated scores about health in general, major health outcomes (current treatment for diabetes, stroke), and various non-specific symptoms of health (sleep quality, headaches) as well as socio-demographic (sex, age, marital status) and lifestyle (alcohol consumption, smoking, physical activity) factors. Respondents were requested to assess their health status on a categorical scale which was transformed into a binary variable (0 = “very good” and “good”; 1 = “fair”, “bad” and “very bad”) and used as an indicator of general health status as described in the methodological manual of the European Health Interview Survey [20]. We additionally used the von Zerssen 24 item list of somatic complaints [21] such as tiredness, loss of appetite, abdominal pain, cold feet; these are not specific to any diseases and can therefore be used for broad patients groups or, as in this study, for a population to estimate HRQOL. For each participant, answers to all 4-point Likert scale questions have been summed resulting in a continuous score ranging from 0 (no health complaints) to 96 (maximum health complaints). Mental health was assessed using the mental health section of the SF-36 questionnaire [22], which is an indicator used for evaluating individual patients health status. We recalculated the norm-based score for each participant, where high values reflected low mental health. Respondents had to state their feeling of nervousness, depression, relaxation, demoralisation and happiness on a five point scale. Sleep disturbances were assessed using the sleep disturbance score from the Swiss Health Survey 2007 [23] which addresses difficulties to fall asleep, troubled sleep, frequency of spontaneous awakening, and waking up too early in the morning.

### 2.2. Noise Annoyance and Noise Exposure

Noise annoyance at home due to road traffic, trains, aircrafts, industry and neighbours was evaluated using a four-points Likert scale with categories “no”, “slight”, “considerable”, and “heavy” [24].

Noise exposure assessment was conducted using the same procedure described elsewhere [12]. In brief, the Swiss Federal Statistical Office provided geocodes for each respondent address. Both geocodes were provided for participants who moved between the baseline and follow-up (n = 65). Based on their geocodes, noise exposure was assigned from one of two available models depending on whether study participants resided in Basel-City (urban) or in Basel-Country (suburban). In Basel-City we used a road traffic noise cadaster provided by the Basel-City Office for the Environment and Energy. It is based on a detailed 3D city model that was developed by the land surveying office using photogrammetrically analysed aerial photographs. The road traffic data were derived from a traffic model from the year 2008 [12]. In Basel-Country, values were derived from the nationwide SonBASE model [25,26]. Respondents were assigned average traffic noise values for the day (L_day _06:00–22:00) and the night (L_night _22:00–6:00). Time-weighted daily average noise levels L_dn_ were calculated for rail and road traffic noise including a 10 dB(A) penalty for the nighttime [27]. Values were censored at 30 db(A), and 10 dB(A) increments of L_dn _were used in the analysis. In order to rule out selection bias, exposure values extracted for the geocodes of participants and non-participants were compared.

### 2.3. Statistical Analysis

Baseline and follow-up survey data were combined and analysed with multivariable mixed-effects regression models with random intercept, clustered at the level of the individual to investigate the association between annoyance to each noise source, noise exposure, and the health indicators. The relationships with the von Zerssen symptom score and the SF-36 mental health score were analysed using linear regression, while logistic regression was used for self-reported health status. All models were adjusted for age, age as quadratic polynomial, sex, physical activity (frequency of exercise-induced sweating per week), smoking (current smoker *vs*. non or former smoker), education level (low, middle, high), and marital status (single, married, divorced/widowed). A further adjustment was conducted to account for urban *vs*. suburban region, where the two different noise models (3D city model *vs.* SonBASE) have been used.

In order to evaluate potential effect modification, stratified analyses and interaction tests with annoyance to noise source or noise exposure were conducted by sex, age (subjects aged below and above median = 47 years), noise exposure level (subjects exposed below and above median = 46 dB(A)), BMI (cut-off value = 25), and sleep disturbance score from the Swiss Health Survey 2007 [23] (subjects below and above median = 5.61, where individuals scoring higher than median had the most sleep disturbances). A further stratification was conducted for self-reported doctor-diagnosed comorbidity, defined as suffering two or more diseases (arthritis, bronchitis, myocardial infarction, stroke, kidney disease, cancer, osteoporosis or diabetes).

### 2.4. Structural Equation Model (SEM)

**Figure 1:**
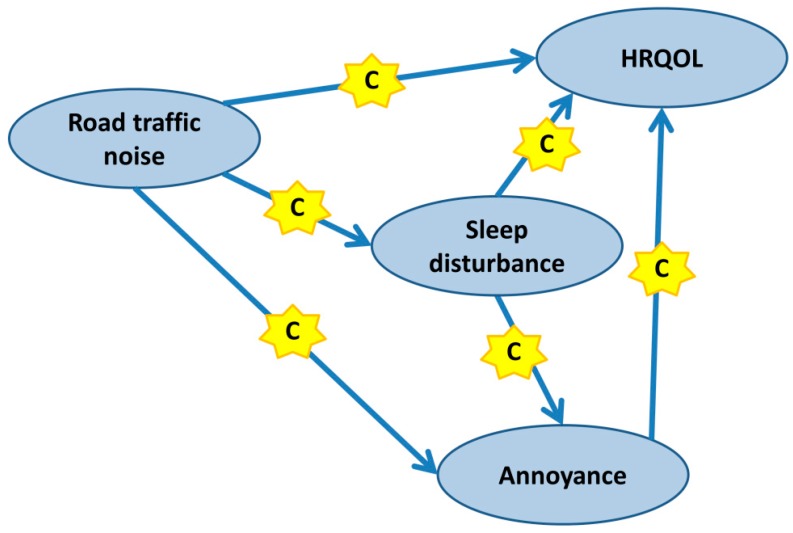
Theoretical model used for the construction of subsequent SEMs for the relationships between road traffic noise, sleep disturbance, annoyance to road traffic noise and HRQOL. The “C” indicates additional factors (confounders) relevant for an association.

Upon identification of sleep disturbance as the main effect modifier, a structural equation model was built to explore the interdependencies between the variables road traffic noise, annoyance to road traffic noise, sleep disturbance and HRQOL. SEM allows for gathering in-depth knowledge on the direct and indirect effects variables may have on each other. As displayed in Figure 1, we specified the SEM in sequential steps based on the literature focussing on the relationships (1) road traffic noise → HRQOL, (2) road traffic noise → sleep disturbance, (3) road traffic noise → annoyance to road traffic noise, (4) sleep disturbance → HRQOL, (5) sleep disturbance → annoyance to road traffic noise and (6) annoyance to road traffic noise → HRQOL. We then built two distinct SEMs for each HRQOL indicator (von Zerssen and SF-36 score) by incrementally increasing their complexity. Relationships (1), (2), (4) and (6) were adjusted for gender, age, physical activity, smoking and education, while relationships (3) and (5) were adjusted for gender, age, urban/suburban and awareness about environmental issues (e.g., fear from car exhaust, sceptical to new technologies) [28]. All variables were z-normalised to obtain comparable regression coefficients. We ran a separate model for baseline and follow-up data. Missing values were excluded yielding 1307/1357 baseline and 1064/1074 follow up observations for SEMs including the von Zerssen/SF-36 mental health indicator. In subsequent steps, non-significant exogenous/endogenous and endogenous/endogenous relationships between variables were constrained to zero. Search for missing paths was conducted using modification indices, and significant paths consistent with the direction of effect were added to the model. Model selection was based on χ^2^, Aikaike Information Criterion (AIC), Tucker-Lewis, Root Mean Squared Error of Approximation (RMSEA) and Standardized Root Mean squared Residuals (SRMR) values. Statistical analyses were carried out using STATA version 13.0 (StataCorp, College Station, TX, USA).

## 3. Results

Out of 3743 eligible study participants, 1375 individuals participated in the baseline investigation (participation rate of 37%) and, of these, 1122 (82%) returned a follow-up questionnaire one year later accounting for a total of 2497 observations. The socio-demographic characteristics of the study sample are displayed in Table 1.

**Table 1:** Socio-demographic characteristics of the 2497 observations.

Age Categories	In %
30–34 Years	13.3
35–39 Years	13.5
40–44 Years	17.7
45–49 Years	17.7
50–54 Years	18.0
>55 Years	19.9
**Sex**	**In %**
Female	59.1
Male	40.9
**Educational level**	**In %**
Low (primary school)	5.9
Medium (apprenticeship)	48.4
High (higher education)	45.7
**Lifestyle characteristics**	
Mean BMI (SD)	24.2 (4.2)
Smokers (%)	27.3
Comorbidity * (%)	11.5

Note: * At least two chronic diseases in the same subject (see text).

**Figure 2:**
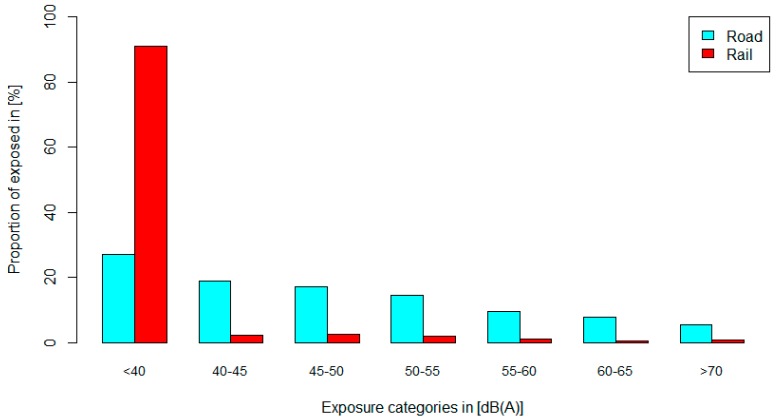
Proportion of the study sample in relation to L_dn_.

In terms of potential selection bias, road traffic and rail noise exposure was not significantly different between participants (mean L_dn_ road: 52.02 ± 6.18 dB(A) and mean L_dn_ railway: 23.59 ± 10.44 dB(A)) and non-participants (52.45 ± 6.28 dB(A) and 24.67 ± 11.10 dB(A)). Figure 2 shows the proportion of the study sample exposed to road and rail noise in 5 dB(A) L_dn_ categories. We decided not to conduct analysis on modelled noise exposure to rail noise due to the small number of highly exposed persons (94% and 95% exposed to L_day_ and L_night_ noise levels <40 dB(A), respectively).

**Figure 3:**
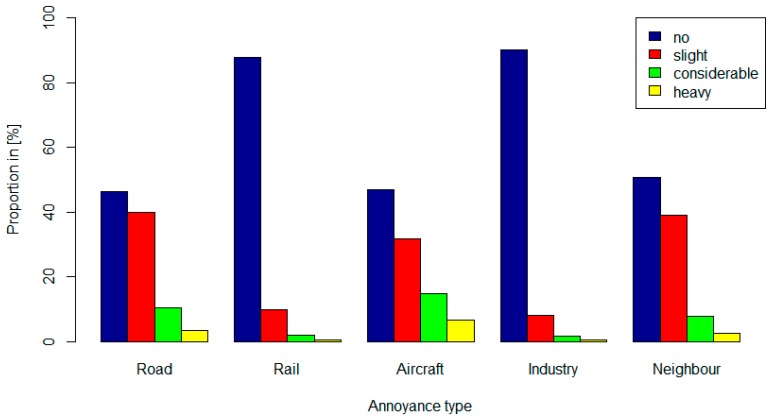
Proportion of the level of annoyance due to different noise sources for the study sample.

Figure 3 shows the distribution of annoyance to various noise sources. The proportion of respondents that reported considerable and heavy annoyance was highest in relation to aircraft noise (21.4%), road traffic noise (13.8%) and neighbour noise (10.2%) and less so for rail (2.4%) and industry noise (1.9%). Univariable regression parameters for annoyance to neighbour noise were found to be strongly associated with annoyance to road (0.21, 95% CI: 0.17, 0.25) and industry (0.17, 95% CI: 0.09, 0.25) noise.

**Figure 4:**
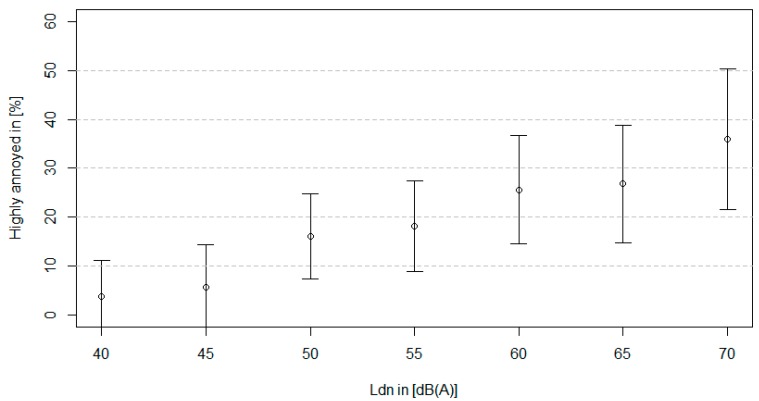
Proportion of the study sample highly (considerable + heavy) annoyed in relation to road traffic noise levels.

Figure 4 shows the relationship between modelled road traffic noise and annoyance. The proportion of the study sample highly (considerable + heavy) annoyed by road traffic noise reaches 36% at an L_dn_ of 70 dB(A).

Crude and adjusted regression parameters for the von Zerssen symptom and SF-36 mental health score are displayed in Table 2.

**Table 2:** Crude and adjusted increase of the von Zerssen symptom score and the SF-36 mental health score in relation to modelled noise (per 10 dB(A) Ldn) or source specific annoyance (per rating category).

Von Zerssen	β [95% CI] Crude	*p*-Value	β [95% CI] Adjusted *	*p*-Value
Road traffic noise 10 dB(A)	0.59 [0.09, 1.09]	0.02	0.47 [−0.01, 0.95]	0.05
Annoyance road	1.50 [1.06, 1.94]	<0.001	1.53 [1.09, 1.96]	<0.001
Annoyance rail	1.03 [0.22, 1.84]	0.01	0.84 [0.06, 1.63]	0.04
Annoyance aircraft	0.76 [0.35, 1.18]	<0.001	0.73 [0.33, 1.14]	<0.001
Annoyance industry	2.14 [1.30, 2.97]	<0.001	2.36 [1.54, 3.17]	<0.001
Annoyance neighbour	1.61 [1.16, 2.07]	<0.001	1.62 [1.17, 2.06]	<0.001
**SF-36 Mental Health**	**β [95% CI] Crude**	***p*−Value**	β [95% CI] Adjusted *	***p*-Value**
Road traffic noise 10 dB(A)	0.47 [−0.05, 0.98]	0.08	0.09 [−0.43, 0.61]	0.73
Annoyance road	1.16 [0.66, 1.66]	<0.001	1.03 [0.54, 1.52]	<0.001
Annoyance rail	1.49 [0.60, 2.37]	<0.01	1.22 [0.34, 2.10]	0.01
Annoyance aircraft	0.12 [−0.33, 0.58]	0.60	0.21 [−0.25, 0.67]	0.37
Annoyance industry	2.16 [1.22, 3.10]	<0.001	2.20 [1.27, 3.12]	<0.001
Annoyance neighbour	1.47 [0.96, 1.98]	<0.001	1.34 [0.83, 1.84]	<0.001

Note: * Adjusted for age, age^2^, sex, physical activity, smoking, education, marital status, region.

After adjusting for covariates, a 10 dB(A) increase of the road traffic noise L_dn_ was associated with a 0.47 (95% CI: −0.01, 0.95) point increase of the von Zerssen symptom score. A substantial increase in the von Zerssen symptom score for annoyance to road, industry and neighbour noise was observed (>1.5 per unit increase in annoyance rating category), while the link with annoyance to railway and aircraft noise was weaker (<1 point per unit increase).

After adjusting for covariates the SF-36 mental health score was not associated with road traffic noise, whereas it was positively associated with most annoyance types with the exception of annoyance to aircraft noise (Table 2). In the crude and adjusted logistic regression models presented in Table 3, self-reported health status was strongly associated with road traffic noise and annoyance to road traffic and neighbour noise. Annoyance to neighbour noise was positively associated with health indicators in all models.

**Table 3:** Crude and adjusted odds ratio for decrease of self-reported health status in relation to modelled noise (per 10 dB(A) Ldn) or source specific annoyance (per rating category).

Self-Reported Health Status	OR [95% CI] Crude	*p*-Value	OR [95% CI] Adjusted *	*p*-Value
Road traffic noise 10 dB(A)	1.36 [1.19, 1.55]	<0.001	1.28 [1.12, 1.48]	<0.001
Annoyance road	1.52 [1.32, 1.77]	<0.001	1.45 [1.25, 1.70]	<0.001
Annoyance rail	1.22 [0.95, 1.58]	0.12	1.07 [0.83, 1.40]	0.58
Annoyance aircraft	0.98 [0.85, 1.13]	0.78	0.99 [0.86, 1.15]	0.96
Annoyance industry	1.43 [1.11, 1.88]	0.01	1.28 [0.97, 1.68]	0.08
Annoyance neighbour	1.79 [1.52, 2.08]	<0.001	1.75 [1.49, 2.08]	<0.001

Note: * Adjusted for age, age^2^, sex, physical activity, smoking, education, marital status, noise model used.

To test effect modification, stratified analyses were conducted for gender, age, noise exposure levels, BMI, sleep disturbance score and occurrence of comorbidity. In general, we found little indication that these factors act as effect modifiers. Sleep disturbance, however, was found to modify the relationship between road traffic noise and the von Zerssen score (*p*-value < 0.001), annoyance to aircraft noise and von Zerssen score (*p*-value = 0.017), annoyance to industry noise and the von Zerssen score (*p*-value < 0.01), and annoyance to neighbour noise and the von Zerssen score (*p*-value < 0.01). These associations were stronger for those people who had a higher sleep disturbance score. Stratified analysis conducted for the SF-36 mental health score and the self-reported health status yielded no results and is therefore not shown.

**Figure 5:**
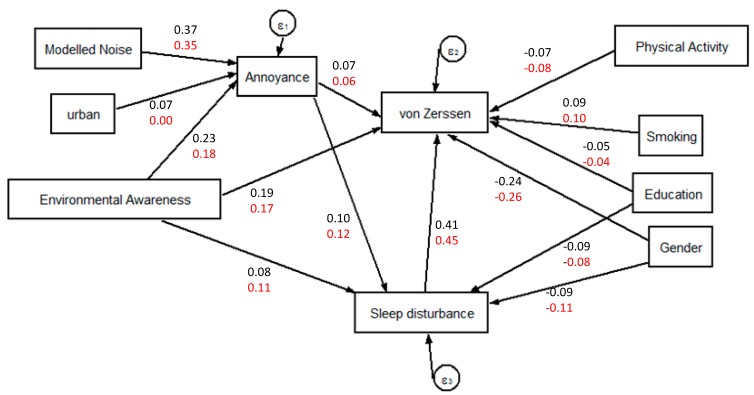
Model A, SEM describing the relation between road traffic noise, annoyance to road traffic noise, sleep disturbance, the von Zerssen score and their confounders. Z-normalised model parameters based on baseline (displayed in black) and follow up (in red) observations.

Figure 5 shows the final SEM (Model A) and the Z-normalised parameters for the relationships between road traffic noise, annoyance to road traffic noise, sleep disturbance, the von Zerssen score and their confounders. Separate models for the baseline and the follow-up data yielded equivalent results. The assumed direct relationship between road traffic noise and the von Zerssen score lost significance with the addition of paths between the von Zerssen and other explanatory variables. Path parameters between road traffic noise and annoyance to road traffic noise, and between sleep disturbance and the von Zerssen score display the highest values.

The path estimates and model fit indices for Model A are shown in Table 4. Both baseline and follow up subsets of Model A have low and non-significant χ^2^ test values indicating a good fit of the model parameters with the observed covariance matrix. Other model fit indices confirm this diagnostic.

**Table 4:** Estimated parameters of SEM, 95% confidence intervals and *p*-values for all relationships and model fit indices for baseline and follow up observations in Model A.

Relationship	Baseline (n = 1307)		Follow up (n = 1064)	
	β [95% CI]	*p*-Value	β [95% CI]	*p*-Value
**Direct effects**				
Road traffic noise → Annoyance to road traffic	0.37 [0.32, 0.42]	<0.001	0.35 [0.29, 0.40]	<0.001
Degree of urban → Annoyance to road traffic	0.07 [0.02, 0.12]	0.007	0.00 [−0.05, 0.06]	0.929
Environmental Awareness → Annoyance to road traffic	0.23 [0.18, 0.28]	<0.001	0.18 [0.12, 0.23]	<0.001
Annoyance to road traffic → von Zerssen	0.07 [0.02, 0.11]	0.003	0.06 [0.01, 0.11]	0.021
Sleep disturbance → von Zerssen	0.41 [0.36, 0.45]	<0.001	0.45 [0.40, 0.50]	<0.001
Environmental Awareness → von Zerssen	0.19 [0.15, 0.24]	<0.001	0.17 [0.12, 0.22]	<0.001
Physical activity → von Zerssen	−0.07 [−0.11, −0.02]	0.002	−0.08 [−0.13, −0.03]	0.003
Smoking → von Zerssen	0.09 [0.04, 0.13]	<0.001	0.10 [0.05, 0.15]	<0.001
Education → von Zerssen	−0.05 [−0.10, −0.01]	0.022	−0.04 [−0.09, 0.01]	0.097
Gender → von Zerssen	−0.24 [−0.28, −0.19]	<0.001	−0.26 [−0.31, −0.21]	<0.001
Annoyance to road traffic → Sleep disturbance	0.10 [0.05, 0.15]	<0.001	0.12 [0.06, 0.18]	<0.001
Environmental Awareness → Sleep disturbance	0.08 [0.03, 0.14]	0.003	0.11 [0.05, 0.17]	<0.001
Education → Sleep disturbance	−0.09 [−0.15, −0.04]	0.001	−0.08 [−0.14, −0.02]	0.011
Gender → Sleep disturbance	−0.09 [−0.15, −0.04]	0.001	−0.11 [−0.17, −0.05]	<0.001
**Indirect effects**				
Annoyance to road traffic → von Zerssen	0.04 [0.02, 0.06]	<0.001	0.05 [0.03, 0.08]	<0.001
Road traffic noise → von Zerssen	0.04 [0.02, 0.06]	<0.001	0.04 [0.02, 0.06]	<0.001
Degree of urban → von Zerssen	0.01 [0.00, 0.01]	0.022	0.00 [−0.01, 0.01]	0.929
Environmental Awareness → von Zerssen	0.06 [0.03, 0.08]	<0.001	0.07 [0.04, 0.10]	<0.001
Education → von Zerssen	−0.04 [−0.06, −0.01]	0.001	−0.03 [−0.06, −0.01]	0.011
Gender → von Zerssen	−0.04 [−0.06, −0.01]	0.001	−0.05 [−0.08, −0.02]	<0.001
Road traffic noise → Sleep disturbance	0.04 [0.02, 0.06]	<0.001	0.04 [0.02, 0.06]	<0.001
Degree of urban → Sleep disturbance	0.01 [0.00, 0.01]	0.031	0.00 [−0.01, 0.01]	0.929
Environmental Awareness → Sleep disturbance	0.02 [0.01, 0.04]	0.001	0.02 [0.01, 0.03]	0.001
**Model fit indices**	**Baseline**		**Follow up**	
χ^2^	3.62		13.42	
*p*-value χ^2^	0.963		0.201	
RMSEA	0.000		0.018	
AIC	36278		28878	
Tucker-Lewis	1.017		0.989	
SRMR	0.006		0.014	

**Figure 6:**
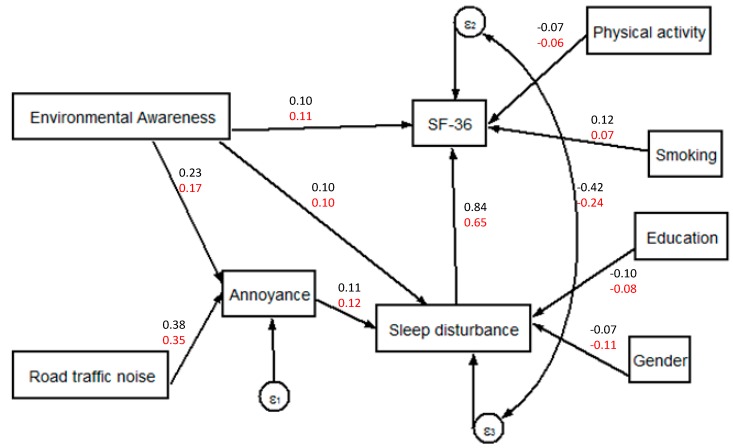
Model B, SEM describing the relation between road traffic noise, annoyance to road traffic noise, sleep disturbance, the SF-36 mental health score and their confounders. Z-normalised model parameters based on baseline (displayed in black) and follow up (in red) observations.

Figure 6 shows the final SEM (Model B) and the Z-normalised parameters for the relationships between road traffic noise, annoyance to road traffic noise, sleep disturbance, the SF-36 mental health score and their confounders. Again, there was no direct relationship between road traffic noise and the SF-36 mental health score upon addition of paths better explaining the latter variable. In contrast to Figure 5, no significant path between annoyance to road traffic noise and the SF-36 mental health score was identified. An indirect link between road traffic noise and SF-36 was found via annoyance to road traffic noise and sleep disturbance.

The path estimates and model fit indices for Model B are shown in Table 5. Both baseline and follow up subsets of Model B have low and non-significant χ^2^ test values indicating a good fit of the model parameters with the observed covariance matrix. Other model fit indices confirm this diagnostic.

**Table 5:** Estimated parameters of SEM, 95% confidence intervals and* p*-values for all relationships and model fit indices for baseline and follow up observations in Model B.

Relationship	Baseline		Follow_up	
	β [95% CI]	*p*-Value	β [95% CI]	*p*-Value
**Direct effects**				
Road traffic noise → Annoyance to road traffic	0.38 [0.33, 0.43]	<0.001	0.35 [0.30, 0.41]	<0.001
Environmental Awareness → Annoyance to road traffic	0.23 [0.18, 0.28]	<0.001	0.17 [0.12, 0.22]	<0.001
Sleep disturbance → SF-36	0.84 [0.54, 1.14]	<0.001	0.65 [0.35, 0.95]	<0.001
Environmental Awareness → SF-36	0.10 [0.04, 0.17]	0.003	0.11 [0.04, 0.18]	0.002
Physical activity → SF-36	−0.07 [−0.11, −0.02]	0.004	−0.06 [−0.12, −0.01]	0.025
Smoking → SF-36	0.12 [0.08, 0.17]	<0.001	0.07 [0.02, 0.13]	0.011
Annoyance to road traffic → Sleep disturbance	0.11 [0.06, 0.16]	<0.001	0.12 [0.06, 0.18]	<0.001
Environmental Awareness → Sleep disturbance	0.10 [0.04, 0.15]	<0.001	0.10 [0.05, 0.16]	<0.001
Education → Sleep disturbance	−0.10 [−0.15, −0.05]	<0.001	−0.08 [−0.14, −0.02]	0.007
Gender → Sleep disturbance	−0.07 [−0.12, −0.02]	0.007	−0.11 [−0.17, −0.06]	<0.001
**Indirect effects**				
Annoyance to road traffic → SF-36	0.09 [0.05, 0.14]	<0.001	0.08 [0.04, 0.12]	<0.001
Road traffic noise → SF-36	0.04 [0.02, 0.06]	<0.001	0.03 [0.01, 0.05]	0.001
Environmental Awareness → SF-36	0.10 [0.04, 0.17]	0.001	0.08 [0.03, 0.13]	0.003
Education → SF-36	−0.09 [−0.13, −0.04]	<0.001	−0.05 [−0.09, −0.01]	0.015
Gender → SF-36	−0.06 [−0.1, −0.02]	0.006	−0.07 [−0.12, −0.03]	0.002
Road traffic noise → Sleep disturbance	0.04 [0.02, 0.06]	<0.001	0.04 [0.02, 0.07]	<0.001
Environmental Awareness → Sleep disturbance	0.03 [0.01, 0.04]	<0.001	0.02 [0.01, 0.03]	0.001
**Model fit indices**				
χ^2^	3.724		10.094	
p-value χ^2^	0.959		0.432	
RMSEA	0.000		0.003	
AIC	33991		26379	
Tucker-Lewis	1.018		1.000	
SRMR	0.006		0.012	

## 4. Discussion

In our analysis using multiple linear models, modelled road traffic noise exposure was strongly associated with self-reported health status but not with the SF-36 mental score and borderline significant with the von Zerssen symptom score. The associations with noise annoyance tended to be stronger and more consistent for all three health indicators, although the pattern was more pronounced for annoyance from road, industry or neighbour noise than for annoyance from railway and aircraft noise.

The SEMs revealed no direct associations linking modelled road traffic noise to the von Zerssen symptom score and the SF-36 mental health score. This finding is in line with previous work [14] where the link between modelled road traffic noise and health outcomes vanished after inclusion of additional variables. However, for both HRQOL indicators we could demonstrate the existence of an indirect path via annoyance and sleep disturbances in both surveys (baseline and follow-up). These indirect paths indicate that annoyance and sleep disturbance act as a mediator for the association between noise exposure and health related quality of life. Interestingly, no direct relationship between annoyance and the SF-36 mental health score was found. According to the work of Stansfeld [29] such a direct relationship may have been observable when including the noise sensitivity in the model, since noise sensitivity affects the psyche and annoyance. Unfortunately, this information is not available in our study.

In both SEMs, the path linking road traffic noise to sleep disturbance vanished after inclusion of the variables education and gender. This is in line with a previous analysis conducted on the present cohort [12] that found no association between road traffic noise exposure and subjective sleep quality. However, a significant association was found between road traffic noise and objective sleep parameters measured by actimetry. The lack of association between road traffic noise and subjective sleep quality implies that people may not be aware of the objective effect of noise on their sleep. This is of particular relevance for research looking at the link between noise and cardiovascular diseases. This further raises the question of the accuracy of annoyance as an indicator for the most severe health effects of noise. 

The von Zerssen score is a HRQOL indicator which, to the best of our knowledge, has not yet been used in noise research. Although we used different health indicators our study results are comparable with previous research on this topic [2,7,8,9]. The direction and magnitude of the observed associations are consistent with the theoretical framework of Soames *et al.* [11]. This demonstrates that noise annoyance and sleep disturbances play important mediating roles for noise induced effects on HRQOL. Our SEMs confirm the statements made by different authors that HRQOL is more closely correlated with reaction and coping of noise exposure than with the physical noise exposure itself [10,11]. The mediator effect of annoyance indicates that both individual coping behaviour and the real noise exposures are important, at least for a common source like road traffic. Conversely, hidden factors triggering annoyance may explain why the proportion of persons highly (considerable and heavy) annoyed by aircraft noise is substantially higher than for any other noise source (Figure 2), although exposure to aircraft noise is relatively low in our study area. According to noise contour maps from the Federal Office of Civil Aviation, no subject in our study sample lived in area with noise ratings [27] L_r, day_ exceeding 57 dB(A) [30] and L_r, 23:00-24:00_ exceeding 47 dB(A), whereas 19 percent of the study sample is exposed to road traffic above 57 dB(A). Such a high annoyance to aircraft noise could, for instance, be explained by increased awareness to this particular noise source through the controversies on the night traffic bans. This phenomenon for example has been previously observed in Switzerland with respect to shooting noise, where only a low correlation with actual exposure values was observed [3]. Alternatively the few aircraft operations taking place between 23:00 and 24:00 at Basel airport may be triggering annoyance because they are well observable due to the generally low background noise levels. Yet, the weaker association observed between annoyance to aircraft noise and the three health scales could be attributable to lower aircraft noise exposure or show that high annoyance does not necessarily translate into a decrease of HRQOL.

Exposure to industry and railway noise is also expected to be low in our sample although modelling data to confirm this was only available for railway noise. Contrary to aircraft noise, for both of these sources the proportion of annoyed persons is also low. Nevertheless, the associations of the three health indicators with railway noise annoyance and with industry noise annoyance are quite different, with considerably stronger associations for the latter indicating that annoyance from a specific noise sources is mediated by additional factors.

We investigated whether the low response rate of 37% could lead to bias in our analysis. We found similar noise exposure of non-respondents compared to respondents, ruling out bias for the relationship between road traffic noise and HRQOL. In terms of annoyance, it was not possible to undertake a non-responder analysis thus it is conceivable that more environmentally concerned people have taken part in this study yielding to an overestimation of the proportions of annoyed people. However, associations between annoyance and HRQOL would only be biased if these people also differ in terms of HRQOL.

Potential limitations when dealing with self-reported annoyance and health outcomes include information bias and confounding. People more susceptible to all kinds of environmental and other factors may express more noise annoyance and more symptoms. We adjusted for relevant confounding factors which, in most cases, decreased the association indicating that residual confounding still might play a role, although unlikely to explain the full association. However, in these regards, the absence of adjustment for noise sensitivity and possible exposure misclassification (façade insulation, location of the bedroom and window opening/closing behaviour could not be considered) is a shortcoming of this study. The cross-sectional analysis also did not allow us to address the timing issue; which comes first, the increase in annoyance, the sleep disturbance or the decrease in HRQOL? As in other studies, we did not have the possibility to assess the proportion of people who moved out of noisy areas because of annoyance. Although our analysis is based on a cohort study, a longitudinal analysis was not possible since only about 65 subjects, those who moved in the one year between baseline and follow-up, had a change of exposure between baseline and follow-up. We further saw no significant difference in the L_dn_ values for participants that moved between baseline and follow-up indicating that self-selection is not expected to play a major role.

## 5. Conclusions

This study demonstrates that sleep disturbances and annoyance play an important role for the effects of road traffic noise on HRQOL.
